# Trends in Sensors Fault Diagnosis

**DOI:** 10.3390/s21062224

**Published:** 2021-03-23

**Authors:** Piotr Witczak

**Affiliations:** Faculty of Computer, Electrical and Control Engineering, Institute of Control and Computation Engineering, University of Zielona Gora, 65-417 Zielona Gora, Poland; p.witczak@issi.uz.zgora.pl

Recently, the automation of processes has been widely demanded. Many industries seek optimization in production time, costs or scrap. One may consider here only a few highly automated branches, such as silicone chips production, waste and water management, and fluid transportation, but due to the recent pandemic, also other benefits in automatic control. However, to fully utilize its potential, reliable and fast fault detection is necessary (either for predictive maintenance or fault-tolerant control). This Special Issue of *Sensors* tries to depict current trends in fault diagnosis in industrial applications. Wide coverage of branches, as well as plurality of recent developments, were the main reasons to compose it. Starting at fault detection for pneumatic valves, through sensors networks, autonomous vehicles (either ground or aerial), water pipelines to much more common gearboxes, roll bearings or brakes. All of those exciting topics are covered within this Special Issue, in all cases bringing new perspective to fault diagnosis applications.

The authors of [1] incorporate the autoregressive neural network with exogenous inputs for behavioral prediction in fault detection and diagnosis of pneumatic valves. IT enables the creation of the signature matrix and the decision tree. Using soft computing methods brings more and more satisfying results as the quality of methods increases in parallel with computational power required in the process. The Kinsman method is also used in [2] for fault detection and fault-tolerant control of magnetic brake system. In this case, however, a neural network is used to tune the state-space model of a system, increasing the quality of more conventional residual-based fault detection mechanism operating with feedback controller. Additionally, [3] incorporates a shallow artificial neural network to scan-chains (using failure feature vectors). The mentioned papers utilize shallow networks, whereas in [4], the authors implement a deep network in form of a stacked autoencoder for the fault diagnosis of roll bearings. Similarly, in [5], the authors present convolutional a deep neural network to classify the potential faults. Some analytical model-based approaches are also in this area of interest. Closer examination of [6], as well as [7], may bring the reader to a conclusion that especially linear models are worth studying. In the case of [6], this is a simple, but powerful linearization around an operating point, whereas in [7], the authors propose a Takagi–Sugeno fuzzy model composed of a number of linear sub-models. While similar in modeling approaches, both papers deal with fault diagnosis in a slightly different way. The authors of the first utilize the well-known Kalman filters and the authors of the latter implement a form of modified Luenberger observer. The above-mentioned approaches share some similarities and thus advantages and disadvantages, but also differ in many ways. One must carefully study both to pick a suitable solution or idea and adapt it for their own advantages.

In constrast to the above mentioned, the authors of [8] focus their efforts in enhancing the quality of frequency-based methods in fault diagnosis. They propose data enhancement for subsequent wavelet transform. The fault is then detected by an SVM classifier. The main idea for fault detection and performance degradation prediction in [9] is also based on the Support Vector Machine. However, the authors proposed a heavily modified algorithm in the form of kernel function hybrid, optimized by krill herd. Here, as in [8], the methods are applied in the frequency domain. The aforementioned SVM is also a part of the fault diagnosis method proposed in [10], where the one-against-one multiclass support vector machine is fed by a signal with noise reduced by using the adaptive noise filtering technique. The noise reduction method is based on the LMS filter with Gaussian reference noise generator. This very interesting technique is applied in the frequency domain.

Finally, [11] compares a magnitude of approaches on fault detection, isolation, identification and even recovery for automotive sensors, such as LIDAR. The authors compare 120 papers from the last 15 years and give a comprehensive insight into the undertaken topic. The authors explicitly classify faults of perception sensors into seven categories, provide exemplary methods to deal with them and review them. Additionally, some sub-classes are considered. One of the interesting outcomes of this survey is a conclusion that, recently, most researchers are focused on environmental-related faults, such as rain, fog or snow. This seems valid and reasonable for elucidating whether weather conditions are most the common source of faults and noises, as they cannot be controlled.

Comparing the proposed approaches, it becomes clear that there is no universal solution for all fault-diagnosis problems; rather, a solution needs to be fitted to the particular case. All mentioned papers clearly show that this is the best approach. Considering either analytical or soft-computing methods, one must first deeply understand an underlying system and pick a most suitable method. The editor has a deep hope that this short Special Issue on sensors fault diagnosis will help many readers choose their path and discover solutions fitted to their needs.
